# Demystifying the pathway of assessment and treatment for bipolar disorder – utilising co-production and algorithms to personalise the approach

**DOI:** 10.1192/bjo.2021.421

**Published:** 2021-06-18

**Authors:** Jessica Nicholls-Mindlin, Digby Quested, Matthew Taylor, Lauren Fuzi, David Gee

**Affiliations:** 1 University of Oxford; Angus McLellan, Oxford Health NHS Foundation Trust; 2 Department of Psychiatry, University of Oxford,Oxford Health NHS Foundation Trust; 3 Oxford Health NHS Foundation Trust

## Abstract

**Aims:**

To develop an evidence based, patient centred treatment pathway for people experiencing symptoms of bipolar disorder (BD), modifiable to include local resources.

**Method:**

This project was developed in line with current approaches to service development such as coproduction, with patient and public involvement (PPI) and enhancing personalisation of treatment in medicine. As part of a local initiative, a multi-disciplinary team was brought together to understand and analyse the current local pathway for those affected by BD. It was found that the approach to assessment and management was not consistent between locality teams. Two experts by experience who have a diagnosis of BD were invited to become involved with the development of the pathway. Meetings were set up to enable coproduction and elicit information from those with the diagnosis. The responses provided insight into the effectiveness of different approaches used nationally to inform the methods and resources that are most helpful and appropriate to comprehensively support those with the illness.

NICE guideline evidence was used to create two algorithms to streamline the care of those with BD in both primary and secondary care. These algorithms include pharmacological, psychological and social approaches. It also considers the junctions at which referrals should be made and the criteria on which decisions are based.

**Result:**

One algorithm was designed for use in primary care and will be distributed to local GPs to clarify the initial steps for assessment and management of BD and the criteria for referral. A second decision tree will be made available to all doctors working in mental health services with detailed medication options, when they are appropriate and whether additional psychological intervention should be considered e.g. post-discharge groups. Other specialist options such as Early Intervention for Psychosis and Perinatal Mental Health Services were also included. An information pack was created to be offered to all those with a diagnosis or possible diagnosis of BD. This contains useful resources such as skills and exercises that patients may find of benefit, external resources and websites regarding additional support and further information on BD, its nature and management.

**Conclusion:**

The approach and resources collated here will help to streamline the management of those with bipolar disorder whilst also ensuring a more consistent approach. The involvement of experts by experience and the incorporation of NICE guidelines ensures a well-rounded and comprehensive set of documents that will be helpful to both clinicians and patients.

